# Metagenomic study of the gut microbiota associated with cow milk consumption in Chinese peri-/postmenopausal women

**DOI:** 10.3389/fmicb.2022.957885

**Published:** 2022-08-16

**Authors:** Bo Tian, Jia-Heng Yao, Xu Lin, Wan-Qiang Lv, Lin-Dong Jiang, Zhuo-Qi Wang, Jie Shen, Hong-Mei Xiao, Hanli Xu, Lu-Lu Xu, Xiyu Cheng, Hui Shen, Chuan Qiu, Zhe Luo, Lan-Juan Zhao, Qiong Yan, Hong-Wen Deng, Li-Shu Zhang

**Affiliations:** ^1^School of Physical Science and Engineering, College of Life Sciences and Bioengineering, Beijing Jiaotong University, Beijing, China; ^2^Department of Endocrinology and Metabolism, The Third Affiliated Hospital of Southern Medical University, Guangzhou, China; ^3^Center for System Biology, Data Sciences, and Reproductive Health, School of Basic Medical Science, Central South University, Changsha, China; ^4^Tulane Center for Bioinformatics and Genomics, School of Public Health and Tropical Medicine, Tulane University, New Orleans, LA, United States; ^5^Shunde Hospital of Southern Medical University (The First People’s Hospital of Shunde), Foshan, China

**Keywords:** cow milk consumption, metagenomic, gut microbiota, functional module, network

## Abstract

Cow milk consumption (CMC) and alterations of gut bacterial composition are proposed to be closely related to human health and disease. Our research aims to investigate the changes in human gut microbial composition in Chinese peri-/postmenopausal women with different CMC habits. A total of 517 subjects were recruited and questionnaires about their CMC status were collected; 394 subjects were included in the final analyses. Fecal samples were used for studying gut bacterial composition. All the subjects were divided into a control group (*n = 248*) and a CMC group (*n = 146*) according to their CMC status. Non-parametric tests and LEfSe at different taxonomic levels were used to reveal differentially abundant taxa and functional categories across different CMC groups. Relative abundance (RA) of one phylum (*p_Actinobacteria*), three genera (*g_Bifidobacterium*, *g_Anaerostipes*, and *g_Bacteroides*), and 28 species diversified significantly across groups. Specifically, taxa *g_Anaerostipes* (*p < 0.01*), *g_Bacteroides* (*p < 0.05*), *s_Anaerostipes_hadrus* (*p < 0.01*), and *s_Bifidobacterium_pseudocatenulatum* (*p < 0.01*) were positively correlated with CMC levels, but *p_Actinobacteria* (*p < 0.01*) and *g_Bifidobacterium* (*p < 0.01*) were negatively associated with CMC levels. KEGG module analysis revealed 48 gut microbiome functional modules significantly (*p < 0.05*) associated with CMC, including *Vibrio cholerae* pathogenicity signature, cholera toxins (*p = 9.52e-04*), and cephamycin C biosynthesis module (*p = 0.0057*), among others. In conclusion, CMC was associated with changes in gut microbiome patterns including beta diversity and richness of some gut microbiota. The alterations of certain bacteria including *g_Anaerostipes* and *s_Bifidobacterium_pseudocatenulatum* in the CMC group should be important for human health. This study further supports the biological value of habitual cow milk consumption.

## Introduction

Milk and dairy products are frequently included as important elements in a healthy and balanced diet, which can provide the necessary energy and nutrients to ensure proper growth and development (Pereira, 2014). Epidemiologic studies confirmed the nutritional importance of milk in the human diet and reinforce the possible role of its consumption in preventing several chronic conditions like cardiovascular diseases (CVDs), osteoporosis (Ilesanmi-Oyelere and Kruger, 2020), some forms of cancer, obesity, and diabetes (Pereira, 2014). Milk also provides a multitude of proteins with anti-inflammatory properties, and these bioactive factors may attenuate intestinal inflammation (Chatterton et al., 2013; Thai and Gregory, 2020).

The human gut is populated by trillions of microorganisms, known collectively as the gut microbiota (GM) (Ley et al., 2008). Many human disorders are associated with gut microbiota, such as gastrointestinal disorders like inflammatory bowel disease (IBD) (Odamaki et al., 2016), colorectal cancer (Rao et al., 2006), irritable bowel syndrome, and gastrointestinal cancer (Botteri et al., 2008); and metabolic diseases like diabetes mellitus (Cardinali et al., 2020) and obesity (Cuevas-Sierra et al., 2019). The immune homeostasis of the host is also influenced by gut microbiota (Mason et al., 2015). Gut microbiota can be modulated positively or negatively by different lifestyle and dietary factors (Gilbert et al., 2018).

Several previous studies have assessed the effect of dairy products on human GM, but many have focused on fermented dairy like yogurt (Link-Amster et al., 1994; Odamaki et al., 2012, 2016; Yılmaz et al., 2019; Jie et al., 2021) or special dairy products like casein powder (Beaumont et al., 2017) or whey bars (Reimer et al., 2017). Some recent studies showed that cow milk could influence the alpha (Fernandez-Raudales et al., 2012; Shuai et al., 2021) and beta diversity (Aslam et al., 2021; Shuai et al., 2021) of GM and relative abundance (RA) of some taxa including phyla *p_Bacteroidetes* and *p_Proteobacteria* and genus *g_Roseburia*, *g_Lactobacillus*, *g_Prevotella*, *g_Bifidobacterium*, *g_Clostridium*, *g_Streptococcus*, *g_Atopobium*, *g_ Leuconostoc*, and *g_Veillonella* in humans (Fernandez-Raudales et al., 2012; Bendtsen et al., 2018; Swarte et al., 2020; Aslam et al., 2021; de Carvalho et al., 2022). However, very few studies have reported cow milk’s effect on human gut bacterial species (Aslam et al., 2020). In addition, most of the studies were based on animal models or randomized controlled trials. Population-level observational studies which could really evaluate the effects of habitual dairy intake on the gut microbiome were very rare (Aslam et al., 2021). Women in the postmenopausal stage usually suffered from many kinds of diseases, and some of them including osteoporosis and breast cancer were reported to be associated with gut microbiota (Zhu et al., 2018; Gatti and Fassio, 2019; Ozaki et al., 2021; Rettedal et al., 2021). In this study, fecal samples from Chinese peri-/postmenopausal women were collected and metagenomic shotgun sequencing was performed. Metagenomic analyses were conducted to identify the effect of cow milk consumption (CMC) on GM patterns, including some general compositional features and abundance of each taxon at the phylum, genus, and species levels. In particular, KEGG functional modules associated with CMC were also detected. To our knowledge, this is the first gut microbiome-CMC association study based on shotgun sequencing performed in Chinese peri-/postmenopausal women.

## Materials and methods

### Study subjects and questionnaire

This study was approved by the Third Affiliated Hospital of Southern Medical University (Guangzhou City, China) and performed under the principle of the Helsinki Declaration II. A cohort of 517 unrelated healthy Chinese peri-/postmenopausal women were recruited from June 2014 to January 2018 from local communities. Each subject completed a questionnaire that collected their CMC status and other basic information like age, weight, medication, alcohol drinking, diet, smoking history, and use of nutrition supplements. Specifically, participants selected the CMC from predetermined categories: none (0 ml/day), 250 ml/day, 500 ml/day, and 750 ml or more/day. Subjects were divided into a control group and a CMC group (milk intake ≥ 250 ml/day) according to the questionnaire. The cow milk was pasteurized. Two inclusion criteria were applied in this study: (1) aged 40 years or older and (2) had lived in Guangzhou City for more than 3 months. Exclusion criteria included: (1) Used antibiotics, oestrogens, anticonvulsant, or proton pump inhibitor medications in the past 3 months; (2) Underwent hysterectomy or bilateral ovariectomy; (3) Diabetes mellitus, except for easily controlled, non-insulin-dependent diabetes mellitus; (4) Chronic renal disease manifest by serum creatinine > 1.9 mg/dL; (5) Chronic liver diseases; (6) Significant chronic lung disease; (6) Alcohol abuse; (7) Corticosteroid therapy and anticonvulsant therapy; (8) Other metabolic diseases or inherited bone diseases; (9) major gastrointestinal disease including lactose intolerance; and (10) Any other disease, treatment, or condition that would be an apparent non-genetic factor underlying the variation of BMD, etc. A more detailed exclusion criteria were presented in Supplementary Table 1.

### Fecal sampling and DNA extraction

We collected stool samples from all subjects. The fecal samples were frozen at -80°C within 30 min of sample procurement and used for GM DNA extraction with the E.Z.N.A.^®^ Stool DNA Kit (Omega, Norcross, GA, United States). We stored the GM DNA samples at -80°C until further analyses.

### Metagenomic shotgun sequencing

A fecal DNA library was constructed with the TruSeq Nano DNA LT Library Preparation Kit (FC-121-4001, Illumina, San Diego, CA, United States). The fecal DNA was fragmented by dsDNA Fragmentase (NEB, M0348S, Massachusetts, United States) and incubated at 37°C for 30 min. Fragmented cDNA was used to construct libraries. Blunt-end DNA fragmentation and size selection were performed with provided sample purification beads. An A-base was added to the blunt ends of each strand for the preparation of ligation to indexed adapters. These adapters also contained sequencing primer hybridization sites for single, paired-end, and indexed reads. The ligated products were amplified with polymerase chain reaction (PCR) under the following conditions: initial denaturation at 95°C for 3 min, followed by 8 cycles of 98°C for 15 s (denaturation), 60°C for 15 s, 72°C for 30 s (extension), and then a final elongation at 72°C for 5 min.

Metagenomic shotgun sequencing was performed by LC-Bio Technologies (Hangzhou) CO., LTD. (Hangzhou, China^1^) *via* Hiseq 4000 (Illumina, San Diego, CA, United States) and PE150 strategy. The relative abundance (RA) of unigenes for each sample was estimated by transcripts per kilobase million (TPM, Formula 1, where k was the k*^th^* unigene, r was the number of unigene reads, and L indicated unigene length) based on the number of aligned reads and the unigene length by Bowtie2 v2.2.0.


$$G_{k}=\frac{r_{k}}{L_{k}}\times \frac{1}{\sum_{i=1}^{n}\frac{r_{i}}{L_{i}}}\times 10^{6}\left(1\right)$$

### Bioinformatics

#### Raw data processing

Raw sequencing read data were processed in the following steps: (1) Cutadapt v1.9. was used to remove sequencing adapters from sequencing reads. (2) Low-quality reads were trimmed using Fqtrim v0.94. A sliding window (size = 6 bp) was set to calculate the average quality of the bases within this window, and the 3’ end of reads was trimmed when the average quality value was smaller than 20; we also discarded the reads when the length was less than 100 bp and the percentage of “N” was larger than 5% after trimming. (3) Read alignment to the host genome was performed by Bowtie2 v2.2.0 and host genomic contamination was removed. Once quality-filtered reads were obtained, they were *de novo* assembled to construct metagenomes for each sample by SPAdes v3.10.0. The coding sequences (CDS) of metagenomic contigs were predicted by MetaGeneMark v3.26. The CDS of all samples were clustered by CD-HIT v4.6.1 to obtain unigenes.

#### Taxonomic and functional annotation of unigenes

Unigenes of all samples were aligned according to the NCBI NR database^2^ using DIAMOND software with the lowest common ancestor algorithm. A protein reference based on the KEGG module dataset^3^ was applied for functional annotation. The sum of RA of unigenes within a module represented the RA of an annotated functional module.

The BLASTP function within DIAMOND was used for unigene alignments. It determines the bit score and expected value (E-value) of the computed alignment, which indicates the alignment quality. We selected the best hit with the highest bit score from all the potential hits (*E-values ≤ 1 × 10^–5^*) as the respective KEGG Orthology (KO) for each unigene. KOs were further mapped to GM-associated functional KEGG modules.

### Statistical analysis

Differences in covariates like age, weight, body mass index (BMI), and calcium consumption between the two groups were checked with a Chi-square test or two independent sample *t*-tests according to variable features; *p < 0.05* was considered to achieve statistical significance.

Data normalization methods total sum scaling (TSS) or centered-log ratio (CLR) were applied, respectively, in different analyses. Metagenome-Assembled Genome (MAG) counts at the phylum, genus, and species levels in each group were calculated within R by the criteria that one feature is taken into account only if its RA is more than 0.01% after TSS data transformation. For further community composition analysis, the data were filtered by several criteria: (1) relative abundance > 0.1%; (2) detection rate within all samples > 10%; and (3) low variance filter for comparative analysis: inter-quantile range (IQR) > 10%. Dominant microbiotas were calculated within each group at different taxonomic levels via R according to their RA.

Alpha and beta diversity, which, respectively, demonstrate within-sample microbiota richness and between-sample dissimilarities, were computed within R via the Vegan package in each group at the phylum, genus, and species levels. For alpha diversity, two indexes (Shannon and Simpson) were computed and Mann–Whitney test was used to assess the difference within groups; *p < 0.05* was considered significant. For beta diversity, dissimilarity distances between samples were assessed by the Bray–Curtis matrix, and Principal Coordinates Analysis (PCoA) and Nonmetric Multidimensional Scaling (NMDS) were used to depict it. Permutational analysis of variance (PERMANOVA) test was used to evaluate the beta diversity difference between two groups, and *p < 0.05* was considered significant.

A classical univariate analysis (Mann–Whitney test) was performed to detect differences in RA of each taxon between the CMC group and the control group; *p*-value *< 0.05* was considered statistically significant. In this analysis, we applied both TSS and CLR data normalization methods. TSS is a traditional approach and has been frequently applied in microbiome analysis; however, it is strongly influenced by highly abundant taxa (Badri et al., 2020). CLR transformation could theoretically avoid the compositional effects of microbiome data (McMurdie and Holmes, 2014).

Linear Discriminant Analysis Effect Size (LEfSe) uses the Kruskal–Wallis test, Wilcoxon Rank Sum test, and Linear Discriminant Analysis (LDA) for microbiome biomarker discovery. For this study, LEfSe was performed given two indispensable situations: (1) threshold on the logarithmic LDA score for discriminative features was equivalent to 2.0; and (2) *p*-value *< 0.05* and FDR-adjusted *p < 0.1*.

R package NetCoMi was applied to depict the profile of the gut microbiota’s association. MAG abundance data and sample data were processed by the phyloseq function in the phyloseq package in R. We selected the 100 taxa with the highest abundance to calculate their association in the gut. CLR data norm method was used and the association was calculated using the SPRING method (Yoon et al., 2019). For the network comparison, the 50 most abundant taxa were chosen. To test the difference in networks between the two groups, the absolute differences in network properties were computed. To depict the overall difference of networks in the two groups, the Jaccard index (*j*) was computed; the smaller the *j* was, the more different the two networks were (Peschel et al., 2021).

For the functional module analysis, Mann–Whitney test was applied to assess the richness differences. *p < 0.05* was significant.

### Validation

An American cohort (*n = 260*) was used to validate the results of our analysis. These subjects were divided into a control group (*n = 130*) and a CMC group (*n = 130*) according to the questionnaire, and diversified GMs between these two groups were identified. The data transformation and statistical analysis methods were the same as for the Chinese cohort.

## Results

### Basic characteristics of study subjects

After getting rid of subjects with missing information about CMC status, a total of 394 subjects were divided into two groups: the control group (*n = 248*) and the CMC group (*n = 146*). All subjects were female and no significant difference was observed in their age distribution (*P = 0.79*). There was no significant difference in physical characteristics like weight (*P = 0.85*) or BMI (*P = 0.858*), or in dietary habits like diet prone (*P = 0.59*), alcohol drinking (*P = 0.787*), or pickled or fermented foods (including yogurt) (*P = 0.432*) across CMC status. However, subjects in the CMC group tend to consume yogurt more compared with the control group (Table 1).

**TABLE 1:** Characteristics of Chinese subjects included in the study.

Variables	Control Group (*n* = 248)	CMC Group (*n* = 146)	*P* value
Female, n (%) *	248 (100)	146 (100)	
Postmenopausal, n (%) *	248 (100)	146 (100)	1.000
Age (years; mean ± SD) ^#^	52.8 ± 2.7	52.9 ± 3.4	0.790
Weight (KG; mean ± SD) ^#^	57.2 ± 8.2	57.3 ± 7.2	0.850
BMI (mean ± SD) ^#^	22.9 ± 3.0	22.9 ± 2.6	0.858
Drinking, n (%) *	27 (10.9)	18 (12.3)	0.787
Exercise, n (%) *	168 (67.7)	110 (75.3)	0.138
Daily Sleep Time (hours; mean ± SD) ^#^	6.62 ± 1.34	6.72 ± 1.31	0.503
Vitamin history, n (%) *	50 (20.2)	40 (27.4)	0.127
Calcium history, n (%) *	103 (41.5)	63 (43.2)	0.835
Tea Drink, n (%) *	101 (40.7)	62 (42.5)	0.816
Red meat intake, < 100g/day: > 100g/day, n (%) *	220 (88.7):28 (11.3)	134 (91.8):12 (8.2)	0.423
Vegetable intake, 250g/day:500g/day: > 750g/day, n (%) *	29 (11.7):102 (41.1): 117 (47.2)	23 (15.8):65 (44.5): 58(39.7)	0.280
Water intake, 0.5–1L/day:1–1.5L/day: > 1.5L/day, n (%) *	92 (37.1): 91 (36.7): 65 (26.2)	46 (31.5): 60 (41.1): 40 (27.4)	0.513
Diet, Meat Prone: Balanced: Vegetarian, n (%) *	21 (8.5):149 (60.0): 78 (31.5)	14 (9.6):80 (54.8): 52(35.6)	0.590
Pickled or Fermented Foods, No: < 3times/week: > 3times/week, *n (%)*	182 (73.4):53 (21.4): 13 (5.2)	114 (78.1):23 (15.8): 9 (6.1)	0.432
Yoghurt Consumption, n (%) *	37 (14.92)	49 (33.56)	< 0.05

*Chi-squared test.

^#^Two independent-sample *t*-test.

### Taxonomic composition of gut microbiota

#### Number of different taxonomies

After being filtered by the criteria RA > 0.01%, the total number of phyla was 59 in both groups, but the total number of genera and the total number of species was slightly lower in the CMC group: There were 2,097 genera and 3,426 species in the control group, whereas there were 2,090 genera and 3,354 species in the CMC group (Table 2).

**TABLE 2:** Bacterial composition in each group at different levels.

	Phylum	Genus	Species
Control Group	59	2097	3426
CMC Group	59	2090	3354

Values in the table indicate the number of phyla, genera, and species, respectively, across all fecal samples after being filtered by relative abundance > 0.01% criteria. CMC, Cow milk consumption.

#### Dominated taxa of gut microbiota in subjects

A total of 214 species belonging to 52 genera and 7 phyla remained after the data processing. The five most prevalent phyla were *p_Bacteroidetes*, *p_Firmicutes*, *p_Proteobacteria*, *p_Actinobacteria*, and *p_Fusobacteria*. Bacteria in these phyla accounted for 80% of the total bacteria. At the genus level, *g_Bacteroides*, *g_Prevotella*, *g_Eubacterium*, *g_Clostridium*, and *g_Faecalibacterium* accounted for 65% of the total bacteria. At the species level, *s_Bacteroides_unclassified*, *s_Bacteroides_vulgatus*, *s_Faecalibacterium_prausnitzzi*, *s_Prevotella_corpi*, and *s_Bacteroidales_unclassified* were most abundant, accounting for about 20% of the total species (Table 3).

**TABLE 3:** Dominated taxa in two groups.

Taxa	Control group (%)	CMC group (%)
*p__Bacteroidetes*	43.593	45.385
*p__Firmicutes*	31.235	30.681
*p__Proteobacteria*	3.609	3.109
*p__Actinobacteria*	0.597	0.553
*p__Fusobacteria*	0.220	0.220
*g__Bacteroides*	38.144	42.886
*g__Prevotella*	9.996	7.541
*g__Eubacterium*	6.007	6.469
*g__Clostridium*	5.257	4.917
*g__Faecalibacterium*	4.623	4.631
*s__Bacteroides_unclassified*	9.809	11.003
*s__Bacteroides_vulgatus*	3.089	3.416
*s__Faecalibacterium_prausnitzii*	3.201	3.240
*s__Prevotella_copri*	2.335	1.810
*s__Bacteroidales_unclassified*	1.647	1.762

Five most abundant taxa and their relative abundance are shown in the table. The data were normalized by the total sum scaling method.

#### Alpha and beta diversity

**FIGURE 1:**
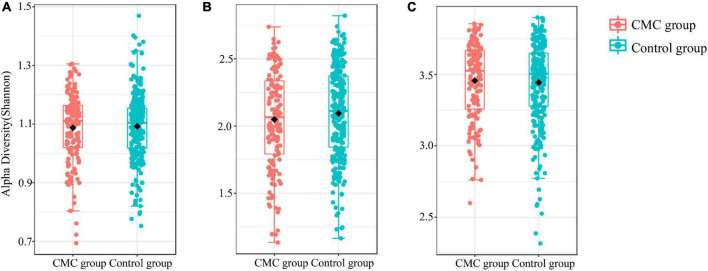
Alpha diversity of subjects grouped by CMC status. The **(A–C)** refer to the Shannon index of gut microbiota at different MAG levels. **(A)** Phylum, **(B)** Genus, **(C)** Species. Data were normalized *via* the TSS method.

**FIGURE 2:**
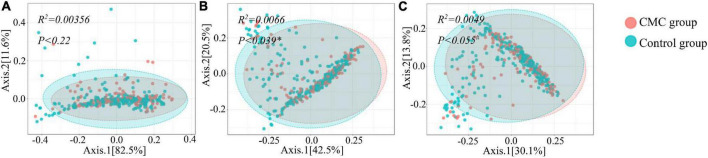
Beta diversity of subjects grouped by CMC status. **(A–C)** depict beta-diversity of gut microbiota according to Bray–Curtis (BC) distance *via* principal coordinates analysis (PCoA) method. **(A)** Phylum, **(B)** Genus, **(C)** Species. Data were normalized via the TSS method, PERMANOVA (Permutational ANOVA) test was applied to detect the difference significance in the two groups and *^#^P < 0.1* or *P < 0.05.

To detect any differences in gut bacterial diversity between the two groups, alpha diversity indexes (Shannon and Simpson) and a beta diversity index (Bray–Curtis distance) were computed. For alpha diversity, no significant difference was observed across the groups at the phylum, genus, or species levels (*P > 0.05*) (Figure 1 and Supplementary Table 2). For beta diversity, at the phylum level, no significant difference was observed (*P_*PCoA*_ < 0.22* and *P_*NMDS*_ < 0.223*) (Figure 2A and Supplementary Table 2); at the genus level, significant *p*-values were achieved (*P_*PCoA*_ < 0.039* and *P_*NMDS*_ < 0.037*) (Figure 2B and Supplementary Table 2); as for the species level, two nearly significant *p*-values were observed (*P_*PCoA*_ < 0.055* and *P_*NMDS*_ < 0.055*) (Figure 2C and Supplementary Table 2).

### Association between gut microbiota and cow milk consumption

We compared the RA of each taxon between two groups *via* Mann–Whitney test at the phylum, genus, and species levels. Data normalization methods total sum scaling (TSS) and centered-log ratio (CLR) were applied, respectively, to minimize the impact of data normalization on the results. For the TSS transformed data analysis, RA of one phylum (*p_Actinobacteria*), three genera (*g__Bifidobacterium*, *g__Anaerostipes, g__Bacteroides*), and 28 species were altered in the CMC group versus the control group (*P < 0.05*) and only two (*g__Bacteroides, P_*CLR*_ = 0.084; s__Prevotella_copri_CAG_164, P_*CLR*_ = 0.057*) of these taxa have not been observed to diversify significantly in the CLR transformed data analysis. The five most significantly diversified taxa completely overlapped for these two data normalization methods: *p_Actinibacteria* (*P_*TSS*_ = 7.02e-4, FDR P_*TSS*_ = 0.0049; P_*CLR*_ = 4.44e-4, FDR P_*CLR*_ = 0.0031*), *g_Bifidobacterium* (*P_*TSS*_ = 7.02e-4, FDR P_*TSS*_ = 0.019; P_*CLR*_ = 4.44e-4, FDR P_*CLR*_ = 0.0115*), *g__Anaerostipes* (*P_*TSS*_ = 7.33e-4, FDR P_*TSS*_ = 0.019; P_*CLR*_ = 8.21e-5, FDR P_*CLR*_ = 0.0043*), *s__Anaerostipes_hadrus* (*P_*TSS*_ = 7.33e-4, FDR P_*TSS*_ = 0.0932; P_*CLR*_ = 8.21e-5, FDR P_*CLR*_ = 0.0176*), and *s__Bifidobacterium_unclassified* (*P_*TSS*_ = 8.71e-4, FDR P_*TSS*_ = 0.0932; P_*CLR*_ = 4.49e-4, FDR P_*CLR*_ = 0.048*) (Table 4).

**TABLE 4:** Gut microbiota associated with cow milk consumption (CMC) *via* Mann–Whitney test at phylum, genus, and species levels.

Taxonomic level	*P*_*TSS*_	FDR *P*_*TSS*_	*P_*CLR*_*	FDR *P*_*CLR*_
**Phylum**				
*p__Actinobacteria*	0.00070225**	0.0049158**	0.00044415**	0.0031091**
**Genus**				
*g__Bifidobacterium*	0.00070225**	0.019068*	0.00044415**	0.011548*
*g__Anaerostipes*	0.00073337**	0.019068*	0.0000821**	0.0042673**
*g__Bacteroides*	0.031533*	0.38963	*0.083658^#^*	0.48336
**Species**				
*s__Anaerostipes_hadrus*	0.00073337**	*0.093206^#^*	0.0000821**	0.017562*
*s__Bifidobacterium_unclassified*	0.00087109**	*0.093206^#^*	0.00044877**	0.048018*
*s__Bacteroides_sp__3_1_23*	0.0041976**	0.27991	0.0052025**	0.18556
*s__Bifidobacterium_pseudocatenulatum*	0.005232**	0.27991	0.0016195**	0.11552
*s__Firmicutes_bacterium_CAG_882*	0.0072482**	0.30944	0.0036398**	0.18385
*s__Prevotella_sp__CAG_1092*	0.0093964**	0.30944	0.00722848**	0.22098
*s__Bacteroides_salyersiae*	0.010122*	0.30944	0.0042957**	0.18385
*s__Bacteroides_ovatus_CAG_22*	0.015228*	0.32567	*0.017047* *	0.28486
*s__Alistipes_sp__CAG_29*	0.016095*	0.32567	*0.010642* *	0.28468
*s__Bacteroides_ovatus*	0.016339*	0.32567	0.039262*	0.28486
*s__Bacteroides_sp__D2*	0.018318*	0.32567	*0.036142* *	0.28486
*s__Bacteroides_finegoldii_CAG_203*	0.02347*	0.32567	*0.015735* *	0.28486
*s__Alistipes_finegoldii*	0.023582*	0.32567	0.015151*	0.28486
*s__Prevotella_sp__CAG_386*	0.024969*	0.32567	0.030745*	0.28486
*s__Sutterella_sp__CAG_351*	0.025507*	0.32567	*0.020654* *	0.28486
*s__Bacteroides_stercoris_CAG_120*	0.025871*	0.32567	0.02895*	0.28486
*s__Bacteroides_sp__CAG_98*	0.033541*	0.33056	*0.046391* *	0.28486
*s__Bacteroides_xylanisolvens*	0.035496*	0.33056	*0.034624* *	0.28486
*s__Bacteroides_dorei_CAG_222*	0.035737*	0.33056	0.039701*	0.28486
*s__Bacteroides_sp__9_1_42FAA*	0.035899*	0.33056	0.03377*	0.28486
*s__Bacteroidales_bacterium_ph8*	0.037464*	0.33056	0.026991*	0.28486
*s__Bacteroides_sp__1_1_30*	0.038397*	0.33056	0.036964*	0.28486
*s__Prevotella_copri_CAG_164*	0.042142*	0.33056	*0.057165^#^*	0.28486
*s__Oscillibacter_sp__ER4*	0.043743*	0.33056	*0.030183* *	0.28486
*s__Firmicutes_bacterium_CAG_65*	0.044708*	0.33056	0.024733*	0.28486
*s__Roseburia_inulinivorans_CAG_15*	0.045198*	0.33056	0.038226*	0.28486
*s__Firmicutes_bacterium_CAG_95*	0.046998*	0.33056	0.044127*	0.28486
*s__Firmicutes_bacterium_CAG_124*	0.049597*	0.33056	0.027181*	0.28486

*P_TSS_* value, P-value of Classical univariate analysis of data transformed by TSS.

*P_CLR_* value, P-value of Classical univariate analysis of data transformed by CLR.

Difference achieved significance of ^#^*P* < 0.1, *P < 0.05, or **P < 0.01.

### Taxonomic biomarkers associated with cow milk consumption identified by linear discriminant analysis effect size

**FIGURE 3:**
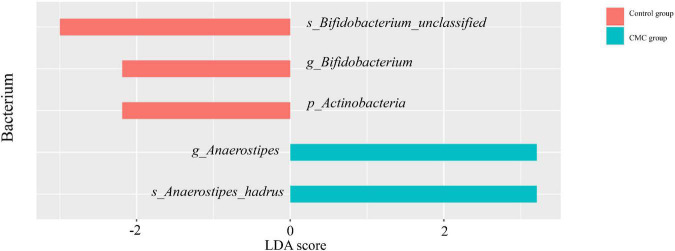
Biomarkers identified by LEfSe. LEfSe indicates differences in the bacterial taxa at different levels (p, phylum; g, genus; s, species), only the taxa having *P < 0.05*, *FDR < 0.1*, and *LDA value > 2* are shown in the figure.

**FIGURE 4:**
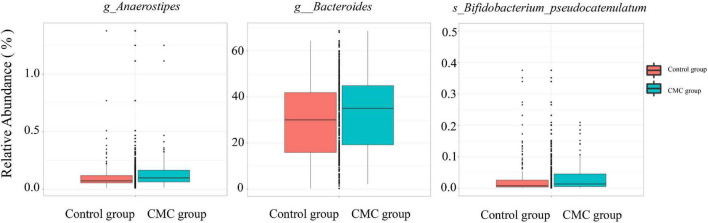
Differences of taxa abundance present in subjects. Data were normalized via total sum scaling (TTS) method and are expressed as relative abundance. Some significantly CMC-associated GM are displayed (g, genus; s, species).

Linear discriminant analysis effect size (LEfSe) analysis was conducted to identify the correlation between gut microbiota and CMC. An LDA score > 0 means the taxa was positively associated with CMC, while an LDA score < 0 indicates a negative association between taxa and CMC. According to the LEfSe results, a total of 36 taxa correlated with CMC (*p < 0.05, | LDA| > 2.0*), but only five of these taxa reached FDR < 0.1. Among them, RA of *p_Actinobacteria* (*FDR = 0.0049, LDA score = −2.19*), *g_Bifidobacterium* (*FDR = 0.019, LDA score = −2.19*), and *s_Bifidobacterium_unclassified* (*FDR = 0.093, LDA score = −3*) decreased in the CMC group, but RA of *g_Anaerostipes* (*FDR = 0.019, LDA score = 3.21*) and *s_Anaerostipes_hadrus* (*FDR = 0.093, LDA score = 3.21*) increased. These taxa were the same as those most significant ones in the Mann–Whitney test. Some other taxa such as *g_Bacteroides* (*p = 0.031*, *LDA = 5.29*) and *s_Bifidobacterium_pseudocatenulatum* (*p = 0.005*, *LDA = 2.92*) were also observed to increase in CMC group, though their FDR > 0.1 (Figures 3, 4 and Supplementary Table 3).

### Yogurt consumption was not associated with cow milk consumption-associated gut microbiota

We have observed some differences in yogurt consumption between the two groups, we further explored its possible association with CMC-associated GM *via* Mann–Whitney test. No significant association was observed and the results were presented in Supplementary Table 4.

### Network construction and comparison

#### Constructing a single microbial interplay network

**FIGURE 5:**
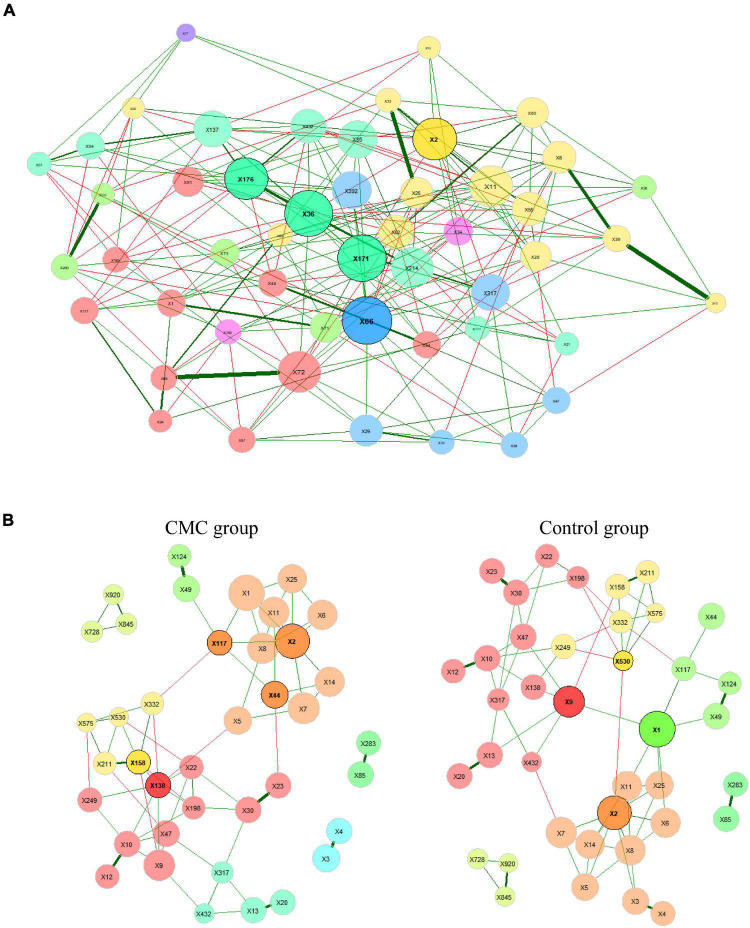
Bacterial associations of samples in two groups. The SPRING method is used as an association measure. The estimated partial correlations are transformed into dissimilarities via the “signed” distance metric and the corresponding (non-negative) similarities are used as edge weights. Green edges correspond to positive estimated associations and red edges to negative ones. Eigenvector centrality is used for defining hubs (nodes with a centrality value above the empirical 95% quantile) and scaling node sizes. Hubs are highlighted by black borders. Node colors represent clusters, which are determined using greedy modularity optimization. The 100 most abundant taxa were analyzed in this part and **(A)** the complete association network where the 50 nodes with the highest degree are shown. **(B)** Comparison of bacterial associations in two groups. Centrality and clustering measures are adopted from the complete network. Species represented by the nodes are given in Supplementary Table 5.

We constructed a taxa network with all the samples studied, and numerous associations between different taxa were observed. The positive edge percentage was 74.61%, which indicated that most of these taxa had positive associations with others. The five hub taxa with the largest empirical quantiles of centralities were *s__Eubacterium_siraeum* (X171), *s__Firmicutes_bacterium_CAG.103* (X176), *s__Bacteroides_unclassified* (X2), *s__Oscillibacter_sp._ER4* (X36), and *s__Roseburia_hominis* (X66). X66 had the highest degree of 23, highest closeness centrality of 0.836, and highest eigenvector centrality of 1.000. Nevertheless, taxa X2 had the highest betweenness centrality (Figure 5A and Supplementary Table 5).

#### Comparing networks between control group and cow milk consumption group

We further constructed the taxa interplay network for the control group and the CMC group and found that the GM’s association diversified across CMC status. First, we compared the hub taxa between the two groups (*j = 0.125*) (Table 5): *s__Ruminococcus_gnavus* (X117), *s__Bacteroides_unclassified* (X2), *s__Clostridium_sp._CAG.7* (X44), *s__Prevotella_copri* (X158), and *s__Roseburia_sp._CAG.18* (X138) were the five most significant hub taxa in the CMC group, but *s__unclassified* (X1), *s__Faecalibacterium_prausnitzii* (X9), *s__Bacteroides_unclassified* (X2), and *s__Prevotella_sp._CAG.386* (X530) were hub taxa in the control group (Figure 5B). Second, as shown in Table 6, the degree, betweenness centrality, closeness centrality, and eigenvector centrality of certain nodes were different across groups. For example, *s__Clostridium_sp._CAG.7* (X44) had a higher degree in the CMC group while *s__Prevotella_sp._CAG.386* (X530) had a higher degree in the control group. Third, the overall characteristics of the two networks also diversified, especially the most central nodes defined regarding betweenness centrality (*j = 0.077*) and closeness centrality (*j = 0.000*) (Table 5). Finally, the network clustering solution was also dissimilar (adjusted Rand index = 0.0157). As shown in Figure 5B, an apparent dissimilarity of the network clustering across groups is that the cluster comprising *s__Bacteroides_stercoris* (X3) and *s__Bacteroides_stercoris_CAG.120* (X4) only existed in the CMC group. In the control group, these two bacteria were assigned to a large cluster connected by the correlation of *s__Bacteroides_stercoris* (X3) with *s__Bacteroidales_unclassified* (X8) and *s__Bacteroides_unclassified* (X2).

**TABLE 5:** Jaccard index values corresponding to the networks shown in Figure 5B.

	*j*	*P(J ≤ j)*	*P(J ≥ j)*
Degree	0.389	0.77674	0.39149
Betweenness centrality	0.077	0.038537	0.995
Closeness centrality	0.000	0.132	1.000
Eigenvec. centrality	0.615	0.991	0.034655
Hub taxa	0.125	0.195	0.961

Index values j express the similarity of the sets of most central nodes and also of the sets of hub taxa between the two networks. “Most central” nodes are those with a centrality value above the empirical 75% quantile. Jaccard’s index is 0 if the sets are completely different and 1 for exactly equal sets. *P* (*J* ≤ *j*) is the probability that Jaccard’s index takes a value less than or equal to the calculated index *j* for the present total number of taxa in both sets and *P* (*J* ≥ *j*) is defined analogously. Jaccard index ranges from 0 (completely different) to 1 (sets equal).

**TABLE 6:** Results from testing global network metrics and centrality measures of the networks in Figure 5B.

	CMC group	Control group	*abs.diff.*
Global network measures:			
Average path length [1]	1.996	1.907	0.089
Clustering coefficient [2]	0.294	0.329	0.035
Modularity [3]	0.486	0.531	0.045
Vertex connectivity [4]	1.000	1.000	0.000
Edge connectivity [5]	1.000	1.000	0.000
Density [6]	0.131	0.118	0.013
Degree [7]:			
*s__Roseburia_sp._CAG.18* (X138)	8	3	5
*s__Clostridium_sp._CAG.7* (X44)	5	1	4
*s__Prevotella_sp._CAG.386* (X530)	4	6	2
*s__Bacteroides_stercoris* (X3)	1	3	2
*s__Bacteroides_dorei* (X14)	3	5	2
Betweenness centrality [8]:			
*s__Prevotella_sp._CAG.386* (X530)	4	113	109
*s__unclassified* (X1)	9	108	99
*s__Bacteroides_unclassified* (X2)	57	153	96
*s__Roseburia_sp._CAG.18* (X138)	98	3	95
*s__Faecalibacterium_prausnitzii* (X9)	34	193	69
Closeness centrality [9]:			
*s__Bacteroides_stercoris* (X3)	2.885	19.713	16.827
*s__Bacteroides_stercoris_CAG.120* (X4)	2.885	16.422	13.537
*s__Prevotella_sp._CAG.386* (X530)	19.32	25.916	6.597
*s__Clostridium_sp._CAG.7* (X44)	21.449	15.646	5.804
*s__unclassified* (X1)	20.144	25.228	5.084
Eigenvector centrality [10]:			
*s__Clostridium_sp._CAG.7* (X44)	0.158	0.010	0.147
*s__Ruminococcus_gnavus* (X117)	0.185	0.052	0.133
*s__Bacteroides_dorei* (X14)	0.171	0.293	0.122
*s__Bacteroides_massiliensis* (X5)	0.108	0.227	0.119
*s__Bacteroides_stercoris* (X3)	0.034	0.150	0.116

Shown are, respectively, the computed measure for CMC group and control group, the absolute difference between groups was computed; for degree, betweenness centrality, closeness centrality, and eigenvector centrality analysis: the five taxa with the highest absolute group difference are shown. Local and global network properties implemented in NetCoMi: [1] Arithmetic mean of all shortest paths between vertices in a network. [2] Proportion of triangles with respect to the total number of connected triples^2^, Expresses how likely the nodes are to form clusters. [3] Expresses how well the network is divided into communities (many edges within the identified clusters and only a few between them). [4][5] Minimum number of edges or vertices (nodes) that need to be removed to disconnect the network, respectively. Not meaningful for a fully connected network. [6] Ratio of the actual number of edges in the network and the possible number of edges. Not meaningful for a fully connected network. [7] Number of adjacent nodes. [8] Fraction of times a node lies on the shortest path between all other nodes. A central node has the ability to connect sub-networks. [9] Reciprocal of the sum of shortest paths between this node and all other nodes. The node with the highest closeness centrality has the minimum shortest path to all other nodes. [10] Calculated via eigenvalue decomposition: Ac = λc, where λ denotes the eigenvalues and c denotes the eigenvectors of the adjacency matrix A. Eigenvector centrality is then defined as the i-th entry of the eigenvector belonging to the largest eigenvalue A node is central if it is connected to other nodes having themselves a central position in the network.

### Cow milk consumption-associated functional modules

**FIGURE 6:**
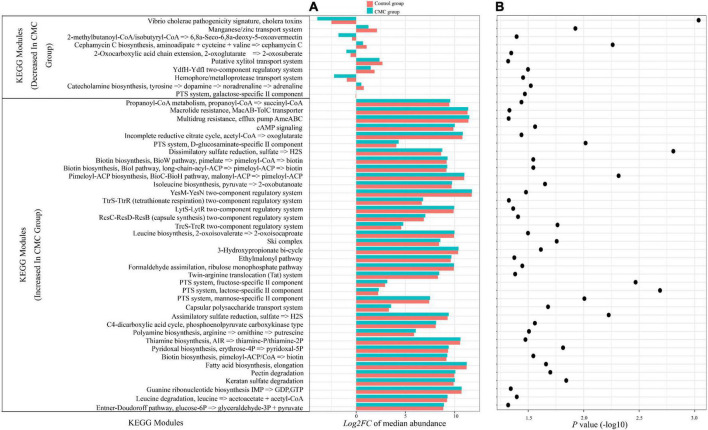
CMC alters the functional potential of the gut microbiome. **(A)** Microbial genes annotated to Kyoto Encyclopedia of Genes and Genomes (KEGG) orthologs (KOs). The bar chart displays the log2 fold change of the relative abundance median of all individual KOs within a module following the Control group (pink bars) or the CMC group (blue bars), respectively. **(B)** Dot plot of the negative log10 of the P-value from the *Mann–Whitney* test of KEGG module abundance of two groups.

A total of 596 KEGG modules were analyzed, and 48 of them diversified significantly (*p* < 0.05) across the groups as detected by the Mann–Whitney test (Figure 6). Of these, 38 significant modules increased in the CMC group, including dissimilatory sulfate reduction (*p* = 0.0016); PTS system, lactose-specific II component (*p = 0.0021*); PTS system, fructose-specific II component (*p = 0.0035*); pimeloyl-ACP biosynthesis (*p = 0.005*), and others. Ten modules, including *Vibrio cholerae* pathogenicity signature, cholera toxins (*p = 9.52e-04*), and cephamycin C biosynthesis module (*p = 0.0057*), among others, decreased in the CMC group (Figure 6).

### Validation

The basic characteristics of the American cohort were shown in Supplementary Table 6. No significant difference in either postmenopausal status (*p = 0.494*) or BMI (*p = 0.484*), or exercise (*p = 0.281*) (Supplementary Table 6) was detected between the CMC group and the control group. Just same as that in the Chinese cohort, the data were normalized via TSS or CLR and LEfSe was used to detect the differential microbiota across groups in the American cohort. As is shown in Table 7, *s__Bacteroides_salyersiae* (*P_1_ = 0.010*, *P_2_ = 0.044*) and *s__Bifidobacterium_pseudocatenulatum* (*P_1_ = 0.002, P_2_ = 0.016*) showed consistent association results in both American cohort and Chinese cohort.

**TABLE 7:** Gut microbiota that showed replicated results in the American cohort.

GM	Data transform method	*P*_1_	*P*_2_	*LDA*
s__Bacteroides _salyersiae	TSS	0.010	0.044	4.12
s__Bifidobacterium _pseudocatenulatum	CLR	0.002	0.016	3.73

P_1_, P-value in Chinese cohort.

P_2_, P-value in American cohort.

LDA, LDA score of LEfSe analysis of American cohort.

TSS, Total sum scaling.

CLR, Centered-log ratio.

## Discussion

Cow’s milk is conventionally considered to be beneficial. In this research, we tried to elucidate the association of CMC with human gut microbiota composition and functional modules, which will be helpful for understanding the possible mechanisms of its effects on human health.

### Cow milk consumption associated with gut microbiotas composition features

We first computed the number of taxa and determined the dominating taxa in the control and CMC groups. The dominated taxa in the two groups were the same, which indicated that CMC may not influence human’s most abundant gut microbiota. We also compared the alpha and beta diversity between groups. Several research have studied the association of CMC with GM diversity; however, their results were inconsistent. Fernandez-Raudales et al. (2012) reported that CMC was negatively associated with alpha diversity; Bendtsen et al. (2018) concluded that there’s no association between CMC and alpha/beta diversity); Aslam et al. (2021) reported that beta diversity of gut microbiota differed among milk consumer and non-consumers. In the current study, we figured that the two groups had different beta diversity at levels of genus and species, while no difference in alpha diversity between groups was found. This was partially consistent with Bendtsen and Hajara Aslam’s results.

### Cow milk consumption associated with the relative abundance of some taxa

Following the analysis of GM general composition features, we detected the association (by Mann–Whitney test) and correlation (by LEfSe) between RA of taxa and CMC status in two cohorts. It was found that the abundance of certain taxa was significantly different between the control group and the CMC group.

### s_Bifidobacterium_pseudocatenulatum, g_Bifidobacterium, and p_Actinobacteria

The abundance of *s_Bifidobacterium_pseudocatenulatum* was positively associated with CMC in both the Chinese and American cohorts (*P < 0.05*, and *LDA > 2* in both cohorts). Such association has not been reported before. It has been reported that a strain of *s_B. pseudocatenulatum* could reverse the adverse effects of diet-induced obesity through the gut-bone axis (Fernández-Murga et al., 2020). Some studies also show an advantage of *s_B.pseudocatenulatum* in treating obesity-associated diseases (Mauricio et al., 2017; Sanchis-Chordà et al., 2019). In addition, *s_B.pseudocatenulatum* was reported to be beneficial for human health as an inflammation regulator (Moya-Pérez et al., 2015; Sanchis-Chordà et al., 2019). It could also ameliorate gut flora dysbiosis, especially the depletion of the SCFA-producing bacteria *Anaerostipes* (Muñoz-Tamayo et al., 2011). In our results, *s_B.pseudocatenulatum* was positively associated with CMC. We hypothesized that CMC is likely to promote human health at least partially through the positive effect of gut microbiota *s_B. pseudocatenulatum*.

*s_B.pseudocatenulatum* belongs to genus *g__Bifidobacterium* (a branch of *p_Actinobacteria*). Of note, in the Chinese cohort, both *p_Actinobacteria* and *g_Bifidobacterium* were detected to be negatively associated with CMC (*FDR < 0.1* and *LDA < −2*). The association of CMC with *p_Actinobacteria* has not been reported before. An explanation for the decrease of *g_Bifidobacterium* observed in the CMC group might be that milk is a provider of calcium since Shili et al. (2020) showed that subjects with reduced calcium consumption had a higher abundance of *g_Bifidobacterium*.

### g_Anaerostipes

An increase in abundance of *g_Anaerostipes* was observed in the CMC group in our research. Although this association had not been replicated in the validation American cohort, it was consistent with Xq. Li.’s conclusion that whole milk consumption significantly increased *Anaerostipes* (*p < 0.01*) (Li et al., 2018). The increase of the bacteria *g_Anaerostipes* in the CMC group may also contribute to intestinal health. Genus *g_Anaerostipes* is a branch of phylum *Firmicutes*, which is a dominant bacterial taxon in the human gut. The anaerobic bacteria *g_Anaerostipes* can produce butyrate from lactic acid (Muñoz-Tamayo et al., 2011; Thomas et al., 2014). Our genomic sequencing data also confirmed the potential butyrate-producing capability of *g_Anaerostipes* since it encoded genes of the “Reductive acetyl-CoA pathway (Wood-Ljungdahl pathway)”. This is an important pathway involved in the biosynthesis of SCFAs butyrate (Portincasa et al., 2022). Butyrate is an SCFA responsible for regulating mucosal gene expression and maintaining gut barrier integrity. It can regulate the release of insulin and glucagon and provide energy for host cells (Hague et al., 1997). Butyrate also inhibits histone deacetylase-induced apoptosis of colon cells and activates gluconeogenesis through a cAMP-dependent mechanism (Davie, 2003). In another study, the *Anaerostipes* genus was associated with a higher estimated glomerular filtration rate in the overall population and non-diabetes mellitus subgroup, which also indicated that *g_Anaerostipes* is beneficial for renal function (Thomas et al., 2014). Species *s__Anaerostipes_hadrus* (*P = 7.32e-4, LDA score = 3.21*), which belongs to *g_Anaerostipes*, were also identified to be positively associated with CMC in our study.

### *s__Bacteroides_salyersiae* and *g_Bacteroides*

Relative abundance (RA) of *s__Bacteroides_salyersiae* diversified in both Chinese and American cohorts (*p < 0.05*). This species belongs to *g_Bacteroides*, which was observed to increase (*p = 0.03, LDA = 5.29*) in the CMC group in the Chinese cohort. This is consistent with a report showing that an increase of *g_Bacteroides* usually results from the long-term intake of protein and animal fat (Wu et al., 2011). *g_Bacteroides* were reported to be beneficial for human health. Aerobic exercise can increase *g_Bacteroides* abundance and thus improve cardiopulmonary function (Morita et al., 2019). It is also an inflammation regulator; a decrease in *g_Bacteroides* level can lead to a decrease in inflammatory cytokines (Fan et al., 2019). Another study has shown that *g_Bacteroides* can activate T cells in the bodies of infants, thereby promoting their immune system development (Walker and Iyengar, 2015).

#### Cow milk consumption may alter gut microbiota association network

We further studied the GM association network in each group via the NetCoMi package to better understand the complex interplay of microbial communities. We compared the networks of subjects with different CMC statuses and found that they were essentially different. We observed different hub taxa in the CMC group compared with the control group, which indicated that CMC may at least partially change the interaction of GM. Hub taxa usually act as connector nodes linking multiple clusters/modules in the network, thus their tiny abundance changes may affect the balance of microbe clusters apparently. By comparing the five centrality measures of each taxon and the network clustering between the CMC and control groups, *s__Bacteroides_stercoris* (X3) attracted our attention. The degree, the closeness centrality, and the eigenvector centrality of *s__Bacteroides_stercoris* all changed a lot across groups. The correlations of *s__Bacteroides_stercoris* with *s__Bacteroides_unclassified* and *s__Bacteroidales unclassified* disappeared in the CMC group, which directly altered the clustering of the network. In this study, the RA of *s__Bacteroides_stercoris* was a little bit higher in the CMC group than the control group, but the difference was not statistically significant. Recently, Gaundal et al. (2022) reported that the abundance of human gut *Bacteroides Stercoris* was associated with higher adherence to Healthy Nordic Food Index (HNFI) and lower diastolic blood pressure. However, the milk intake was not studied in that research. Based on the changes of the five centrality measures (degree, betweenness centrality, closeness centrality, eigenvector centrality, and hub taxa), there were also some other bacteria that should be highlighted. They are s__*Ruminococcus_gnavus* (X117), *s__Faecalibacterium_prausnitzii* (X9), *s__Prevotella_copri* (X158), *s__Clostridium_sp._CAG.7* (X44), *s__Roseburia_sp._CAG.18* (X138), *s__Prevotella_sp._CAG.386* (X530), *and s__Bacteroides_dorei* (X14). Further studies of them, especially those focused on their interplay with other microbes, should be helpful for elucidating CMC’s effects on GM.

#### Cow milk consumption associated with richness of functional categories

Following the GM profile analysis, we sought to identify the varied modules between groups and found that many modules diversified greatly. The most striking result is that the KEGG module M00850 (*Vibrio cholerae* pathogenicity signature, cholera toxins) was decreased in the CMC group (*p = 9.52e-04*). The reduction of M00850 came from the reduced richness of gut pathogenic bacteria carrying genes ctxB and rtxA, which may induce gastroenteritis in humans (Fang et al., 2019). The reduction of M00850 in the CMC group indicated that CMC habit may be good for maintaining the correct balance between helpful bacteria and harmful bacteria. Cephamycin C biosynthesis module also decreased in the CMC group (*p = 0.0057*). According to our metagenome sequencing results, Cephamycin C biosynthesis genes were encoded by the *s__Clostridiales_bacterium, s__Paenibacillus_chitinolyticus, s__Paenibacillus_sp._G4*, and so on. The inhibition of cephamycin C biosynthesis may also be beneficial to gut probiotics, as it is a kind of antibiotic and may affect the balance of intestinal flora.

A total of 38 KEGG biology modules were positively associated with CMC, four of which are correlated to biotin synthesis. Biotin is a B-complex vitamin that acts as an essential coenzyme for five carboxylases. These carboxylases participate in several chemical processes in the cell, including gluconeogenesis, amino acid metabolism, and fatty acid synthesis. Mammals obtain biotin from food, but it can also be produced from gut bacteria (Saleem and Soos, 2022); According to our metagenome sequencing results, biotin synthesis genes were encoded by a lot of microbes such as *s__Anaerostipes_hadrus, s__Escherichia_coli, s__Bacteroides_vulgatus, s__Bacteroides_dorei*, and so on. Biotin regulates immunological and inflammatory functions. It plays a key role in the function of natural killer (NK) lymphocytes and the generation of cytotoxic T lymphocytes (Kuroishi, 2015; Agrawal et al., 2016). Our results indicated that CMC could improve the gut bacteria’s biotin synthesis and thus should be good for host health.

We also observed alterations in the richness of PTS-related modules. Module galactose-specific component decreased in the CMC group while modules mannose-, lactose-, fructose-, and D-glucosaminate-specific II component increased in the CMC group. It was indicated that CMC may influence the proportional composition of gut microbiota which utilize different PTS-related enzymes.

The strengths of the present study include: (1) We applied shotgun sequencing technology and detected the RA of taxa at the species level; (2) The GM sequencing was done in a relatively large cohort and the statistical power of our study was well guaranteed. We did the power assessment with a recently published R package “Powmic” (Chen, 2020), which enables power analysis for metagenomic sequencing case-control study for identifying differentially abundant microbes. For non-parametric Wilcoxon rank sum test, given a nominal FDR level of 0.1, with our dataset (N1 = 248, N2 = 146, and the taxa filtering criteria used in this study), the overall power evaluation results are as follows: empirical statistical Power “true positive rate” TPR[TPR = TP/(TP+FN)] = 0.81, False Positive Rate FPR[FPR = FP/(FP+TP)] < 0.00001; (3) The data processing and filter were strict.

The weaknesses of our research are as follows: (1) All subjects were women, so it is not known whether CMC has similar effects on men; (2) Our research was cross-sectional. Although we have determined the correlations between GM and CMC, further studies are still needed to elucidate its mechanisms; (3) This study does not allow for controlling all potential confounding factors that might have effect on human gut microbiome, which may lead to some statistical artifacts; (4) In this study, we used an American cohort as the validation cohort. There should be great differences between the Chinese cohort and the American cohort in the genetic background of the host, the lifestyles, or maybe the gastrointestinal microbial community environment. This may be one of the reasons why only two differential taxa are being replicated in the American cohort. We encourage researchers to use a validation cohort that is as similar as possible to the discovery cohort.

Overall, we have identified several gut microbiota taxa and modules significantly associated with CMC in the present study. Some of the positively associated differential taxa or functional modules have been reported to have positive effects on humans health. Women at peri/postmenopausal stage usually suffered from many kinds of diseases, such as osteoporosis, breast cancer, and obesity. Further studies are still needed to elucidate the effects and underlying mechanisms of CMC habit on peri/postmenopausal women’s health that is mediated by the gut microbiome.

## Conclusion

The present study has revealed alterations in gut bacterial composition and functional modules associated with CMC. These results suggest that cow milk consumption was associated with the beta diversity and abundance of some beneficial bacterial taxa such as *s__B.pseudocatenulatum*, *g_Anaerostipes*, and *g_Bacteroides*. In addition, cow milk consumption was associated with the abundance of many functional modules such as *Vibrio cholerae* pathogenicity signature, cholera toxins, and biotin synthesis, which further support the biological value of habitual cow milk consumption.

## Data availability statement

The datasets presented in this study can be found in online repositories. The names of the repository/repositories and accession number(s) can be found below: https://www.ebi.ac.uk/ena, PRJEB50761.

## Ethics statement

The studies involving human participants were reviewed and approved by The Medical Ethical Committees of Southern Medical University. The patients/participants provided their written informed consent to participate in this study. Written informed consent was obtained from the individual(s) for the publication of any potentially identifiable images or data included in this article.

## Author contributions

HS, CQ, ZL, and L-JZ: subject enrollment. XL, H-MX, QY, and XC: conceptualization. J-HY and L-DJ: data curation. BT, J-HY, W-QL, and Z-QW: formal analysis. H-WD: funding acquisition. L-DJ and L-LX: investigation. HX: methodology. JS, L-SZ, and H-WD: supervision. BT: writing – original draft. BT, L-SZ, and H-WD: writing – review and editing. All authors contributed to the article and approved the submitted version.

## Footnotes

1
www.lc-bio.com

2
ftp://ftp.ncbi.nlm.nih.gov/blast/db/FASTA/nr.gz

3
https://www.genome.jp/kegg/module.html
