# Supplementary material for: Metagenomic study of the gut microbiota associated with cow milk consumption in Chinese peri-/postmenopausal women
Source: Front Microbiol. 2022 Aug 16;13:957885. doi: 10.3389/fmicb.2022.957885 (PMC9425034; doi:10.3389/fmicb.2022.957885)
Supplement: Supplementary file 1 [file Table_1.DOCX]

Supplementary Table 1 Exclusion criteria for the Chinese subjects

| Number | Exclusion criteria |
| --- | --- |
| 1 | Used antibiotics, oestrogens, anticonvulsant or proton pump inhibitor medications in the past three months; |
| 2 | Underwent hysterectomy or bilateral ovariectomy; |
| 3 | Serious residuals from cerebral vascular disease; |
| 4 | Diabetes mellitus, except for easily controlled, non-insulin dependent diabetes mellitus; |
| 5 | Chronic renal disease manifest by serum creatinine > 1.9 mg/dL; |
| 6 | Chronic liver diseases; |
| 7 | Significant chronic lung disease; |
| 8 | Alcohol abuse as defined by those who drink alcohol regularly and cannot control themselves and become intoxicated at least once a week. |
| 9 | Corticosteroid therapy at pharmacologic levels currently, or for more than 6 months duration at any time; |
| 10 | Treatment with anticonvulsant therapy currently, or for more than 6 months duration at any time; |
| 11 | Evidence of other metabolic diseases or inherited bone diseases such as hyper- or hypoparathyroidism, Paget's disease, osteomalacia, osteogenesis imperfecta, or others; |
| 12 | Rheumatoid arthritis, except for minor cases that involve only hand joint and wrist; |
| 13 | Recent major gastrointestinal disease (within the past year) such as celiac disease, post-gastrectomy, Crohn's disease, ulcerative colitis,　Barrett esophagitis, gastrointestinal cancer, gastroesophageal reflux disease, irritable bowel syndrome, pancreatic diseases, lactose intolerance, gastric ulcer, or others; |
| 14 | Any other disease, treatment (e.g., bisphosphonates), or condition that would be an apparent non-genetic factor underlying the variation of BMD. |
