# Supplementary material for: Metagenomic study of the gut microbiota associated with cow milk consumption in Chinese peri-/postmenopausal women
Source: Front Microbiol. 2022 Aug 16;13:957885. doi: 10.3389/fmicb.2022.957885 (PMC9425034; doi:10.3389/fmicb.2022.957885)
Supplement: Supplementary file 2 [file Table_2.DOCX]

Supplementary Table 2 Comparison of alpha and beta-diversity in two groups

| α-diversity index | Taxonomy | Control Group | CMC Group | *P* value |
| --- | --- | --- | --- | --- |
| Shannon, (mean ± sd) ^*^ | Phylum | 1.092 ± 0.014 | 1.092 ± 0.013 | 0.725 |
|  | Genus | 2.095 ± 0.127 | 2.049 ± 0.122 | 0.285 |
|  | Species | 3.443 ± 0.080 | 3.457 ± 0.080 | 0.743 |
| Simpson, (mean ± sd) ^*^ | Phylum | 0.610 ± 0.005 | 0.606 ± 0.005 | 0.906 |
|  | Genus | 0.763 ± 0.008 | 0.748 ± 0.008 | 0.214 |
|  | Species | 0.904 ± 0.000 | 0.907 ± 0.000 | 0.185 |
|  |  |  |  |  |
| β-diversity depict method | Taxonomy | F_value | R_square | *P* value |
| PCoA^#^ | Phylum | 1.141 | 0.0036 | <0.22 |
|  | Genus | 2.622 | 0.0066 | *<0.039** |
|  | Species | 1.920 | 0.0049 | <0.055 |
| NMDS^#^ | Phylum | 1.413 | 0.0036 | <0.223 |
|  | Genus | 2.622 | 0.0066 | *<0.037** |
|  | Species | 1.920 | 0.0049 | <0.055 |

^*^Mann-Whitney test; ^#^PERMANOVA (Permutational ANOVA) test; Difference achieved significance of **P<0.05*
