# Supplementary material for: Metagenomic study of the gut microbiota associated with cow milk consumption in Chinese peri-/postmenopausal women
Source: Front Microbiol. 2022 Aug 16;13:957885. doi: 10.3389/fmicb.2022.957885 (PMC9425034; doi:10.3389/fmicb.2022.957885)
Supplement: Supplementary file 3 [file Table_3.DOCX]

Supplementary Table 3 LEFSe indicating differences in the bacterial taxa at levels of phylum, genus and species

| Taxonomic levels | *P*values | FDR *P* values | LDA score |
| --- | --- | --- | --- |
| p__Actinobacteria | *0.00070108^**^* | *0.0049076^**^* | -2.19 |
| g__Bifidobacterium | *0.00070108^**^* | *0.019036^*^* | -2.19 |
| g__Anaerostipes | *0.00073215^**^* | *0.019036^*^* | 3.21 |
| g__Bacteroides | *0.031497^*^* | 0.38919 | 5.29 |
| s__Anaerostipes_hadrus | *0.00073215^**^* | *0.093053^#^* | 3.21 |
| s__Bifidobacterium_unclassified | *0.00086966^**^* | *0.093053^#^* | -3 |
| s__Bifidobacterium_pseudocatenulatum | *0.0052246^**^* | 0.27952 | 2.92 |

LDA: Linear discriminant analysis

Difference achieved significance of ^#^*P<0.1* or *^*^P<0.05* or *^**^P<0.01*
