# Supplementary material for: Metagenomic study of the gut microbiota associated with cow milk consumption in Chinese peri-/postmenopausal women
Source: Front Microbiol. 2022 Aug 16;13:957885. doi: 10.3389/fmicb.2022.957885 (PMC9425034; doi:10.3389/fmicb.2022.957885)
Supplement: Supplementary file 4 [file Table_4.DOCX]

Supplementary Table 4 Association between yoghurt consumption and CMC-associated gut microbiota detected by Mann-Whitney test

| Taxa | P | FDR |
| --- | --- | --- |
| p__Actinobacteria | 0.061949 | 0.21682 |
| g__Bifidobacterium | 0.061949 | 0.56567 |
| g__Anaerostipes | 0.98547 | 0.98547 |
| g__Bacteroides | 0.13435 | 0.69977 |
| s__Anaerostipes_hadrus | 0.98547 | 0.99146 |
| s__Bifidobacterium_unclassified | 0.060909 | 0.79719 |
| s__Bifidobacterium_pseudocatenulatum | 0.18596 | 0.80068 |
