# Supplementary material for: Metagenomic study of the gut microbiota associated with cow milk consumption in Chinese peri-/postmenopausal women
Source: Front Microbiol. 2022 Aug 16;13:957885. doi: 10.3389/fmicb.2022.957885 (PMC9425034; doi:10.3389/fmicb.2022.957885)
Supplement: Supplementary file 5 [file Table_5.DOCX]

Supplementary Table 5 Species and their Abbreviations in the network analysis

| OTU | Abb. | OTU | Abb. |
| --- | --- | --- | --- |
| s__unclassified | X1 | s__Clostridium_sp._ATCC_29733 | X301 |
| s__Bacteroides_unclassified | X2 | s__unidentified_virus | X302 |
| s__Bacteroides_stercoris | X3 | s__Prevotella_salivae | X303 |
| s__Bacteroides_stercoris_CAG.120 | X4 | s__Cloacimonetes_unclassified | X304 |
| s__Bacteroides_massiliensis | X5 | s__Clostridium_sp._CAG.62 | X305 |
| s__Bacteroides_uniformis | X6 | s__Lachnospiraceae_bacterium_3_1_46FAA | X306 |
| s__Bacteroides_vulgatus | X7 | s__Lachnospiraceae_bacterium_1_4_56FAA | X307 |
| s__Bacteroidales_unclassified | X8 | s__Clostridium_bartlettii_CAG.1329 | X308 |
| s__Faecalibacterium_prausnitzii | X9 | s__Bacteroides_sp._CAG.661 | X309 |
| s__Roseburia_inulinivorans | X10 | s__Faecalitalea_cylindroides | X310 |
| s__Bacteroides_fragilis | X11 | s__.Eubacterium._dolichum | X311 |
| s__Roseburia_inulinivorans_CAG.15 | X12 | s__Oscillibacter_sp._KLE_1745 | X312 |
| s__Firmicutes_bacterium_CAG.65 | X13 | s__Clostridium_sp._D5 | X313 |
| s__Bacteroides_dorei | X14 | s__Streptococcus_unclassified | X314 |
| s__Bacteroides_sp._CAG.98 | X15 | s__Roseburia_sp._CAG.182 | X315 |
| s__Ruminococcus_sp._CAG.108 | X16 | s__Blautia_unclassified | X316 |
| s__Roseburia_sp._CAG.100 | X17 | s__Clostridium_sp._CAG.127 | X317 |
| s__Alistipes_putredinis | X18 | s__Porphyromonas_sp._31_2 | X318 |
| s__Firmicutes_bacterium_CAG.24 | X19 | s__Enterococcus_faecalis | X319 |
| s__Clostridium_sp._CAG.91 | X20 | s__Clostridium_sp._SY8519 | X320 |
| s__Phascolarctobacterium_sp._CAG.207 | X21 | s__Collinsella_sp._CAG.166 | X321 |
| s__Eubacterium_sp._CAG.76 | X22 | s__Clostridium_unclassified | X322 |
| s__Eubacterium_eligens_CAG.72 | X23 | s__Clostridium_sp._FS41 | X323 |
| s__Clostridiales_unclassified | X24 | s__Ruminococcus_albus | X324 |
| s__Bacteroides_ovatus | X25 | s__Porphyromonadaceae_unclassified | X325 |
| s__Ruminococcus_bromii | X26 | s__.Clostridium._cellulosi | X326 |
| s__Roseburia_unclassified | X27 | s__Sutterella_wadsworthensis | X327 |
| s__Bacteroides_caccae | X28 | s__Firmicutes_bacterium_CAG.424 | X328 |
| s__Roseburia_intestinalis | X29 | s__.Clostridium._innocuum | X329 |
| s__.Eubacterium._eligens | X30 | s__uncultured_bacterium_Contigcl_1787 | X330 |
| s__Alistipes_unclassified | X31 | s__Bifidobacterium_longum | X331 |
| s__Bacteroides_sp._D20 | X32 | s__Prevotella_unclassified | X332 |
| s__Bacteroides_xylanisolvens | X33 | s__Lachnoclostridium_phytofermentans | X333 |
| s__Bacteroides_coprocola | X34 | s__Staphylococcus_aureus | X334 |
| s__Subdoligranulum_variabile | X35 | s__Ruminococcus_unclassified | X335 |
| s__Oscillibacter_sp._ER4 | X36 | s__Lachnospiraceae_bacterium_3_1_57FAA_CT1 | X336 |
| s__Bacteroides_uniformis_CAG.3 | X37 | s__Anaerotruncus_sp._CAG.390 | X337 |
| s__Bacteroides_clarus | X38 | s__Robinsoniella_peoriensis | X338 |
| s__Parabacteroides_distasonis | X39 | s__Eubacterium_rectale_CAG.36 | X339 |
| s__Bacteroides_eggerthii | X40 | s__Thermobrachium_celere | X340 |
| s__Butyricimonas_virosa | X41 | s__bacterium_MS4 | X341 |
| s__Proteobacteria_bacterium_CAG.139 | X42 | s__Blautia_sp._CAG.257 | X342 |
| s__Bacteroides_sp._4_1_36 | X43 | s__Bacteroides_sp._2_1_56FAA | X343 |
| s__Clostridium_sp._CAG.7 | X44 | s__Erysipelotrichaceae_bacterium_6_1_45 | X344 |
| s__Parabacteroides_unclassified | X45 | s__Bacteroides_intestinalis_CAG.564 | X345 |
| s__Faecalibacterium_sp._CAG.82 | X46 | s__Clostridium_bolteae_CAG.59 | X346 |
| s__Eubacterium_rectale | X47 | s__Halobacillus_trueperi | X347 |
| s__Firmicutes_bacterium_CAG.110 | X48 | s__Bacteroides_sp._CAG.530 | X348 |
| s__Escherichia_coli | X49 | s__Streptococcus_agalactiae | X349 |
| s__Alistipes_putredinis_CAG.67 | X50 | s__Butyrivibrio_proteoclasticus | X350 |
| s__Bacteroides_gallinarum | X51 | s__Clostridiales_bacterium_NK3B98 | X351 |
| s__Bacteroides_vulgatus_CAG.6 | X52 | s__Bacteroides_coprocola_CAG.162 | X352 |
| s__Bacteroides_sp._3_1_40A | X53 | s__Odoribacter_laneus | X353 |
| s__Clostridium_sp._CAG.43 | X54 | s__Parvimonas_micra | X354 |
| s__Bacteroides_finegoldii | X55 | s__Firmicutes_bacterium_CAG.212 | X355 |
| s__Bacteroides_intestinalis | X56 | s__Clostridium_sp._CAG.242 | X356 |
| s__Bacteroides_nordii | X57 | s__Enterobacter_cloacae | X357 |
| s__Eubacterium_sp._CAG.86 | X58 | s__Bacteroides_pectinophilus_CAG.437 | X358 |
| s__Eubacterium_sp._CAG.202 | X59 | s__Robinsoniella_unclassified | X359 |
| s__Bacteroides_sp._9_1_42FAA | X60 | s__Firmicutes_bacterium_CAG.194 | X360 |
| s__Bacteroides_sp._4_3_47FAA | X61 | s__Clostridium_sp._CAG.411 | X361 |
| s__Bacteroides_thetaiotaomicron | X62 | s__Clostridium_sp._CAG.277 | X362 |
| s__Bacteria_unclassified | X63 | s__Clostridium_botulinum | X363 |
| s__Peptoclostridium_difficile | X64 | s__Klebsiella_unclassified | X364 |
| s__Clostridium_sp._CAG.122 | X65 | s__Coprococcus_sp._HPP0074 | X365 |
| s__Roseburia_hominis | X66 | s__Candidatus_Alistipes_marseilloanorexicus | X366 |
| s__Ruminococcus_lactaris | X67 | s__Firmicutes_bacterium_CAG.176 | X367 |
| s__Hungatella_hathewayi | X68 | s__Eggerthella_sp._CAG.1427 | X368 |
| s__Eubacterium_unclassified | X69 | s__Eubacterium_sp._CAG.146 | X369 |
| s__Bacteroides_sp._2_2_4 | X70 | s__Ruminococcus_gauvreauii | X370 |
| s__uncultured_bacterium | X71 | s__Erysipelatoclostridium_ramosum | X371 |
| s__Firmicutes_unclassified | X72 | s__Streptococcus_anginosus | X372 |
| s__Bacteroides_sp._3_1_33FAA | X73 | s__Eubacterium_sp._CAG.603 | X373 |
| s__Veillonella_sp._CAG.933 | X74 | s__Dorea_formicigenerans_CAG.28 | X374 |
| s__.Clostridium._clostridioforme | X75 | s__Streptococcus_pneumoniae | X375 |
| s__Blautia_wexlerae | X76 | s__Roseburia_sp._CAG.380 | X376 |
| s__Lachnospiraceae_unclassified | X77 | s__Dorea_longicatena_CAG.42 | X377 |
| s__Bacteroides_cellulosilyticus | X78 | s__Clostridium_sp._7_3_54FAA | X378 |
| s__Lachnospiraceae_bacterium_A4 | X79 | s__Prevotella_sp._CAG.1124 | X379 |
| s__Dorea_formicigenerans | X80 | s__Clostridium_butyricum | X380 |
| s__Blautia_sp._CAG.37 | X81 | s__Ruminococcus_sp._CAG.90 | X381 |
| s__Firmicutes_bacterium_CAG.227 | X82 | s__.Clostridium._methoxybenzovorans | X382 |
| s__Bacteroides_sp._1_1_6 | X83 | s__Desulfovibrio_piger | X383 |
| s__Coprobacillus_sp._CAG.235 | X84 | s__Firmicutes_bacterium_CAG.238 | X384 |
| s__Bacteroides_plebeius | X85 | s__Dielma_fastidiosa | X385 |
| s__Bacteroides_caccae_CAG.21 | X86 | s__Bacteroidales_bacterium_ph8 | X386 |
| s__Bacteroides_thetaiotaomicron_CAG.40 | X87 | s__Bacteroides_sp._CAG.144 | X387 |
| s__Parabacteroides_merdae | X88 | s__Adlercreutzia_equolifaciens | X388 |
| s__Bacteroides_sp._1_1_14 | X89 | s__Prevotella_sp._HJM029 | X389 |
| s__Bacteroides_clarus_CAG.160 | X90 | s__Clostridium_sp._CAG.226 | X390 |
| s__Blautia_sp._CAG.52 | X91 | s__Paenibacillus_sp._P22 | X391 |
| s__Bacteroides_sp._HPS0048 | X92 | s__Ruminococcus_bicirculans | X392 |
| s__Bilophila_wadsworthia | X93 | s__Ruminococcus_sp._CAG.254 | X393 |
| s__Oscillibacter_unclassified | X94 | s__Oribacterium_sp._oral_taxon_078 | X394 |
| s__.Ruminococcus._obeum | X95 | s__Prevotella_sp._CAG.1320 | X395 |
| s__Alistipes_sp._CAG.29 | X96 | s__Clostridium_sp._CAG.590 | X396 |
| s__Eubacterium_ventriosum | X97 | s__Robinsoniella_sp._KNHs210 | X397 |
| s__Eubacterium_desmolans | X98 | s__Fusobacterium_varium | X398 |
| s__Parabacteroides_goldsteinii | X99 | s__Clostridium_sp._CAG.413 | X399 |
| s__Bacteroidetes_unclassified | X100 | s__Bacteroides_coprophilus_CAG.333 | X400 |
| s__Bacteroides_sp._1_1_30 | X101 | s__Escherichia_unclassified | X401 |
| s__.Bacteroides._pectinophilus | X102 | s__Clostridium_sp._CAG.678 | X402 |
| s__Dorea_longicatena | X103 | s__Lactobacillus_ruminis | X403 |
| s__Tyzzerella_nexilis | X104 | s__Bacillus_sp._37MA | X404 |
| s__Parasutterella_excrementihominis | X105 | s__.Clostridium._hylemonae | X405 |
| s__Clostridium_sp._CAG.356 | X106 | s__Erysipelotrichaceae_bacterium_2_2_44A | X406 |
| s__Bacteroides_sp._D22 | X107 | s__Firmicutes_bacterium_CAG.94 | X407 |
| s__Clostridium_sp._CAG.217 | X108 | s__Clostridium_sp._CAG.299 | X408 |
| s__.Ruminococcus._torques | X109 | s__Alistipes_sp._627 | X409 |
| s__Eubacterium_ramulus | X110 | s__Prevotella_sp._CAG.487 | X410 |
| s__.Clostridium._bolteae | X111 | s__Coprobacter_sp._177 | X411 |
| s__Bacteroides_sp._3_1_23 | X112 | s__Eubacterium_sp._CAG.161 | X412 |
| s__Candidatus_Bacteroides_timonensis | X113 | s__Prevotella_histicola | X413 |
| s__Bacteroides_fluxus | X114 | s__Ruminococcus_sp._CAG.177 | X414 |
| s__Blautia_sp._KLE_1732 | X115 | s__Lachnospiraceae_bacterium_8_1_57FAA | X415 |
| s__Parabacteroides_sp._20_3 | X116 | s__Bacillus_cereus | X416 |
| s__.Ruminococcus._gnavus | X117 | s__Lachnospiraceae_bacterium_CAG.215 | X417 |
| s__Pseudoflavonifractor_capillosus | X118 | s__Coprococcus_comes_CAG.19 | X418 |
| s__Ruminococcus_sp._5_1_39BFAA | X119 | s__Pseudobutyrivibrio_ruminis | X419 |
| s__Ruminococcaceae_bacterium_D16 | X120 | s__Blautia_hydrogenotrophica | X420 |
| s__Ruminococcus_sp._CAG.60 | X121 | s__Dysgonomonas_mossii | X421 |
| s__Flavonifractor_plautii | X122 | s__Eubacterium_limosum | X422 |
| s__Parabacteroides_johnsonii | X123 | s__.Clostridium._sporosphaeroides | X423 |
| s__Enterobacteriaceae_unclassified | X124 | s__Clostridium_clostridioforme_CAG.132 | X424 |
| s__Anaerotruncus_colihominis | X125 | s__Clostridium_sp._CAG.632 | X425 |
| s__Bacteroides_dorei_CAG.222 | X126 | s__Capnocytophaga_sp._oral_taxon_332 | X426 |
| s__Ruminococcus_sp._JC304 | X127 | s__Alistipes_finegoldii_CAG.68 | X427 |
| s__Bacteroides_sp._3_1_19 | X128 | s__Butyrivibrio_crossotus_CAG.259 | X428 |
| s__Bacteroides_sartorii | X129 | s__Lactobacillus_crispatus | X429 |
| s__Blautia_sp._CAG.237 | X130 | s__Prevotella_sp._HUN102 | X430 |
| s__Bilophila_sp._4_1_30 | X131 | s__.Clostridium._papyrosolvens | X431 |
| s__Bacteroides_oleiciplenus | X132 | s__Eubacterium_sp._CAG.180 | X432 |
| s__Roseburia_intestinalis_CAG.13 | X133 | s__Bacteroidetes_bacterium_oral_taxon_272 | X433 |
| s__Bacteroides_sp._D2 | X134 | s__Eubacterium_sp._CAG.251 | X434 |
| s__.Eubacterium._hallii | X135 | s__Clostridium_sp._ASF356 | X435 |
| s__Bacteroides_eggerthii_CAG.109 | X136 | s__Holdemania_massiliensis | X436 |
| s__Firmicutes_bacterium_CAG.83 | X137 | s__Firmicutes_bacterium_CAG.272 | X437 |
| s__Roseburia_sp._CAG.18 | X138 | s__Bacteroidaceae_bacterium_MS4 | X438 |
| s__.Clostridium._asparagiforme | X139 | s__Lachnobacterium_bovis | X439 |
| s__Burkholderiales_bacterium_1_1_47 | X140 | s__Erysipelothrix_rhusiopathiae | X440 |
| s__Firmicutes_bacterium_CAG.124 | X141 | s__Bacteroides_sp._CAG.443 | X441 |
| s__.Clostridium._symbiosum | X142 | s__Akkermansia_muciniphila | X442 |
| s__Butyrivibrio_crossotus | X143 | s__Clostridium_sp._CAG.230 | X443 |
| s__Bacteroides_sp._D1 | X144 | s__Ruminococcus_sp._CAG.55 | X444 |
| s__Bacteroides_ovatus_CAG.22 | X145 | s__Clostridium_sp._CAG.1013 | X445 |
| s__Oscillibacter_sp._KLE_1728 | X146 | s__Clostridium_nexile_CAG.348 | X446 |
| s__Bacteroides_salyersiae | X147 | s__Prevotella_marshii | X447 |
| s__Alistipes_onderdonkii | X148 | s__Enterococcus_cecorum | X448 |
| s__Bacteroides_coprophilus | X149 | s__Clostridium_sp._ASBs410 | X449 |
| s__Subdoligranulum_sp._4_3_54A2FAA | X150 | s__Firmicutes_bacterium_CAG.791 | X450 |
| s__Bacteroides_sp._2_1_22 | X151 | s__Lachnospiraceae_bacterium_CAG.25 | X451 |
| s__Parabacteroides_sp._D13 | X152 | s__Lachnospiraceae_bacterium_C6A11 | X452 |
| s__Lachnospiraceae_bacterium_7_1_58FAA | X153 | s__Prevotella_disiens | X453 |
| s__Firmicutes_bacterium_CAG.56 | X154 | s__Oribacterium_sp._P6A1 | X454 |
| s__Alistipes_shahii | X155 | s__Clostridium_sp._CAG.169 | X455 |
| s__Firmicutes_bacterium_ASF500 | X156 | s__Firmicutes_bacterium_CAG.240 | X456 |
| s__Firmicutes_bacterium_CAG.170 | X157 | s__Peptostreptococcaceae_bacterium_OBRC8 | X457 |
| s__Prevotella_copri | X158 | s__Clostridium_sp._01 | X458 |
| s__Parabacteroides_gordonii | X159 | s__Clostridium_sp._CAG.75 | X459 |
| s__Firmicutes_bacterium_CAG.95 | X160 | s__Eubacterium_sp._CAG.786 | X460 |
| s__Oscillibacter_sp._CAG.155 | X161 | s__Bacteroides_fragilis_CAG.558 | X461 |
| s__Bacteroides_sp._CAG.754 | X162 | s__Firmicutes_bacterium_CAG.145 | X462 |
| s__Firmicutes_bacterium_CAG.102 | X163 | s__Ruminococcus_champanellensis | X463 |
| s__Firmicutes_bacterium_CAG.41 | X164 | s__Bacteroides_graminisolvens | X464 |
| s__Clostridium_sp._CAG.81 | X165 | s__Eggerthella_lenta | X465 |
| s__Firmicutes_bacterium_CAG.137 | X166 | s__Parabacteroides_sp._D25 | X466 |
| s__Parasutterella_unclassified | X167 | s__Coprococcus_sp._HPP0048 | X467 |
| s__Coprococcus_catus | X168 | s__Porphyromonas_cangingivalis | X468 |
| s__Coprococcus_eutactus | X169 | s__Prevotella_buccalis | X469 |
| s__Coprococcus_comes | X170 | s__Firmicutes_bacterium_CAG.555 | X470 |
| s__.Eubacterium._siraeum | X171 | s__Lachnospiraceae_bacterium_NK4A144 | X471 |
| s__Propionibacterium_acnes | X172 | s__.Clostridium._saccharogumia | X472 |
| s__Bacteroides_reticulotermitis | X173 | s__Collinsella_unclassified | X473 |
| s__Eubacterium_sp._CAG.248 | X174 | s__Eubacterium_sp._CAG.192 | X474 |
| s__Bacteroides_pyogenes | X175 | s__Eubacterium_sp._CAG.581 | X475 |
| s__Firmicutes_bacterium_CAG.103 | X176 | s__uncultured_Spirochaetales_bacterium_HF0500_06B09 | X476 |
| s__Parasutterella_excrementihominis_CAG.233 | X177 | s__Phocaeicola_abscessus | X477 |
| s__Blautia_producta | X178 | s__Parabacteroides_sp._ASF519 | X478 |
| s__Anaerostipes_hadrus | X179 | s__Ornithobacterium_rhinotracheale | X479 |
| s__Enterococcus_faecium | X180 | s__Lachnospiraceae_bacterium_TWA4 | X480 |
| s__Marvinbryantia_formatexigens | X181 | s__Coprobacillus_sp._3_3_56FAA | X481 |
| s__Roseburia_sp._CAG.471 | X182 | s__Streptococcus_dysgalactiae | X482 |
| s__Eubacterium_plexicaudatum | X183 | s__.Clostridium._celerecrescens | X483 |
| s__Clostridium_sp._CAG.510 | X184 | s__Ruminococcus_sp._CAG.57 | X484 |
| s__Alistipes_timonensis | X185 | s__Lactobacillus_mucosae | X485 |
| s__Eubacterium_sp._CAG.115 | X186 | s__Parabacteroides_sp._CAG.409 | X486 |
| s__Eubacterium_sp._CAG.156 | X187 | s__Dorea_sp._CAG.105 | X487 |
| s__Firmicutes_bacterium_CAG.129 | X188 | s__Prevotella_sp._CAG.732 | X488 |
| s__Tannerella_sp._6_1_58FAA_CT1 | X189 | s__Bacteroides_sp._CAG.20 | X489 |
| s__Butyricimonas_synergistica | X190 | s__Paraprevotella_clara_CAG.116 | X490 |
| s__Firmicutes_bacterium_CAG.882 | X191 | s__Ruminobacter_sp._RM87 | X491 |
| s__Dorea_unclassified | X192 | s__Ruminococcus_gnavus_CAG.126 | X492 |
| s__Lachnospiraceae_bacterium_COE1 | X193 | s__Clostridium_sp._CAG.1024 | X493 |
| s__Holdemania_filiformis | X194 | s__Coprococcus_unclassified | X494 |
| s__Paraprevotella_xylaniphila | X195 | s__Clostridium_sp._HGF2 | X495 |
| s__Alistipes_senegalensis | X196 | s__Youngiibacter_fragilis | X496 |
| s__Alistipes_indistinctus | X197 | s__.Clostridium._cellobioparum | X497 |
| s__Eubacterium_sp._CAG.252 | X198 | s__Odoribacter_splanchnicus_CAG.14 | X498 |
| s__Alistipes_sp._HGB5 | X199 | s__Tannerella_sp._oral_taxon_BU063 | X499 |
| s__Bacteroides_helcogenes | X200 | s__Barnesiella_intestinihominis | X500 |
| s__Bacteroides_faecis | X201 | s__Negativicoccus_succinicivorans | X501 |
| s__.Clostridium._leptum | X202 | s__Lachnospiraceae_bacterium_JC7 | X502 |
| s__Roseburia_sp._CAG.45 | X203 | s__Prevotella_sp._CAG.279 | X503 |
| s__Ruminococcus_sp._CAG.9 | X204 | s__Firmicutes_bacterium_CAG.466 | X504 |
| s__Alistipes_finegoldii | X205 | s__Catenibacterium_mitsuokai | X505 |
| s__Clostridium_sp._KLE_1755 | X206 | s__Paenibacillus_lactis | X506 |
| s__Paraprevotella_clara | X207 | s__.Clostridium._aminophilum | X507 |
| s__Bacteroides_salanitronis | X208 | s__Bacteroides_sp._CAG.1076 | X508 |
| s__Bacteroides_faecichinchillae | X209 | s__Lachnospiraceae_bacterium_2_1_58FAA | X509 |
| s__Bilophila_unclassified | X210 | s__Streptococcus_mitis | X510 |
| s__Prevotella_copri_CAG.164 | X211 | s__Solobacterium_moorei | X511 |
| s__Bacteroides_sp._2_1_33B | X212 | s__Streptococcus_thermophilus | X512 |
| s__Blautia_schinkii | X213 | s__Prevotella_melaninogenica | X513 |
| s__Faecalibacterium_sp._CAG.74 | X214 | s__.Clostridium._termitidis | X514 |
| s__Erysipelotrichaceae_unclassified | X215 | s__Klebsiella_variicola | X515 |
| s__Bacteroides_stercorirosoris | X216 | s__Clostridium_sp._CAG.149 | X516 |
| s__Dorea_sp._AGR2135 | X217 | s__Salmonella_enterica | X517 |
| s__Roseburia_sp._CAG.50 | X218 | s__Anaerovorax_odorimutans | X518 |
| s__Lachnoclostridium_unclassified | X219 | s__Paenibacillus_popilliae | X519 |
| s__Intestinibacter_bartlettii | X220 | s__Sutterella_sp._CAG.351 | X520 |
| s__Ruminococcus_callidus | X221 | s__Paenibacillus_larvae | X521 |
| s__Odoribacter_splanchnicus | X222 | s__Prevotella_brevis | X522 |
| s__Oscillibacter_sp._CAG.241 | X223 | s__Prevotella_sp._CAG.1031 | X523 |
| s__Phascolarctobacterium_succinatutens | X224 | s__Clostridium_sp._CAG.264 | X524 |
| s__Klebsiella_pneumoniae | X225 | s__Shigella_flexneri | X525 |
| s__Anaerostipes_caccae | X226 | s__Oribacterium_sp._FC2011 | X526 |
| s__Clostridium_sp._CAG.465 | X227 | s__Clostridium_sp._KNHs214 | X527 |
| s__Bacteroides_finegoldii_CAG.203 | X228 | s__Peptostreptococcaceae_bacterium_VA2 | X528 |
| s__Barnesiella_viscericola | X229 | s__Prevotella_multiformis | X529 |
| s__Bacteroides_cellulosilyticus_CAG.158 | X230 | s__Prevotella_sp._CAG.386 | X530 |
| s__Veillonella_dispar | X231 | s__Clostridium_sp._CAG.138 | X531 |
| s__Intestinimonas_butyriciproducens | X232 | s__Clostridium_saccharoperbutylacetonicum | X532 |
| s__Bacteroides_acidifaciens | X233 | s__Megamonas_funiformis_CAG.377 | X533 |
| s__Prevotella_bivia | X234 | s__Prevotella_denticola | X534 |
| s__Lachnospiraceae_bacterium_6_1_37FAA | X235 | s__Parabacteroides_johnsonii_CAG.246 | X535 |
| s__Butyricicoccus_pullicaecorum | X236 | s__Bacteroides_sp._CAG.702 | X536 |
| s__Clostridiales_bacterium_1_7_47FAA | X237 | s__Pyramidobacter_piscolens | X537 |
| s__Roseburia_sp._CAG.197 | X238 | s__Lachnospiraceae_bacterium_MC2017 | X538 |
| s__Ruminococcus_obeum_CAG.39 | X239 | s__unidentified_phage | X539 |
| s__.Clostridium._scindens | X240 | s__Alistipes_sp._CAG.268 | X540 |
| s__Roseburia_sp._CAG.303 | X241 | s__Acinetobacter_radioresistens | X541 |
| s__Collinsella_aerofaciens | X242 | s__Oribacterium_sp._NK2B42 | X542 |
| s__Bacteroides_barnesiae | X243 | s__Clostridium_sp._CAG.505 | X543 |
| s__Enterobacter_unclassified | X244 | s__Lachnospiraceae_bacterium_AD3010 | X544 |
| s__Clostridium_leptum_CAG.27 | X245 | s__Candidatus_Soleaferrea_massiliensis | X545 |
| s__Prevotella_sp._BV3P1 | X246 | s__Coprobacter_fastidiosus | X546 |
| s__Clostridium_sp._KNHs209 | X247 | s__Anaerotruncus_sp._CAG.528 | X547 |
| s__Blautia_hansenii | X248 | s__Roseburia_sp._CAG.309 | X548 |
| s__Eubacterium_sp._CAG.38 | X249 | s__Erysipelotrichaceae_bacterium_5_2_54FAA | X549 |
| s__Prevotella_stercorea | X250 | s__.Clostridium._aerotolerans | X550 |
| s__Parabacteroides_sp._HGS0025 | X251 | s__Lachnospiraceae_bacterium_AC2028 | X551 |
| s__.Clostridium._saccharolyticum | X252 | s__.Clostridium._sordellii | X552 |
| s__Oxalobacter_formigenes | X253 | s__Lachnospiraceae_bacterium_6_1_63FAA | X553 |
| s__Butyrivibrio_sp._CAG.318 | X254 | s__Geoalkalibacter_ferrihydriticus | X554 |
| s__Porphyromonas_crevioricanis | X255 | s__Campylobacter_coli | X555 |
| s__Firmicutes_bacterium_CAG.534 | X256 | s__Butyrivibrio_sp._AE2015 | X556 |
| s__Lachnospiraceae_bacterium_A2 | X257 | s__Pseudomonas_sp._CMAA1215 | X557 |
| s__Ruminococcus_flavefaciens | X258 | s__Porphyromonas_gulae | X558 |
| s__Clostridium_sp._ASF502 | X259 | s__.Clostridium._hiranonis | X559 |
| s__Oscillibacter_valericigenes | X260 | s__uncultured_bacterium_52B7 | X560 |
| s__Prevotella_timonensis | X261 | s__Dehalobacter_sp._FTH1 | X561 |
| s__Collinsella_sp._4_8_47FAA | X262 | s__Butyrivibrio_sp._MC2013 | X562 |
| s__Clostridium_sp._CAG.58 | X263 | s__Tannerella_sp._CAG.51 | X563 |
| s__Bacteroides_sp._2_1_16 | X264 | s__Eubacterium_sp._3_1_31 | X564 |
| s__Firmicutes_bacterium_CAG.114 | X265 | s__Oribacterium_asaccharolyticum | X565 |
| s__Alistipes_sp._CAG.53 | X266 | s__Campylobacter_jejuni | X566 |
| s__Lactobacillus_delbrueckii | X267 | s__Lachnospiraceae_bacterium_5_1_57FAA | X567 |
| s__Holdemanella_biformis | X268 | s__Clostridium_cellulovorans | X568 |
| s__Dorea_sp._CAG.317 | X269 | s__Ruminococcus_sp._CAG.330 | X569 |
| s__Streptococcus_salivarius | X270 | s__Tannerella_forsythia | X570 |
| s__.Clostridium._citroniae | X271 | s__Bifidobacterium_pseudocatenulatum | X571 |
| s__Parabacteroides_sp._CAG.2 | X272 | s__Clostridium_beijerinckii | X572 |
| s__Prevotella_maculosa | X273 | s__Sanguibacteroides_justesenii | X573 |
| s__Streptococcus_suis | X274 | s__Bacteroides_sp._CAG.462 | X574 |
| s__Eubacterium_hallii_CAG.12 | X275 | s__Prevotella_sp._CAG.604 | X575 |
| s__Firmicutes_bacterium_CAG.646 | X276 | s__Fluviicola_taffensis | X576 |
| s__Eubacterium_sp._CAG.274 | X277 | s__Lachnospiraceae_bacterium_9_1_43BFAA | X577 |
| s__Bacteroides_sp._CAG.875 | X278 | s__Lachnoanaerobaculum_saburreum | X578 |
| s__Bacteroides_sp._CAG.633 | X279 | s__Coprobacillus_sp._D7 | X579 |
| s__Clostridium_sp._CAG.352 | X280 | s__Lachnospiraceae_bacterium_ND2006 | X580 |
| s__Eubacterium_sp._ER2 | X281 | s__Coprococcus_sp._CAG.131 | X581 |
| s__.Clostridium._methylpentosum | X282 | s__Finegoldia_magna | X582 |
| s__Bacteroides_plebeius_CAG.211 | X283 | s__Butyrivibrio_sp._AC2005 | X583 |
| s__Butyrivibrio_fibrisolvens | X284 | s__Sutterella_sp._CAG.521 | X584 |
| s__Clostridium_sp._CAG.964 | X285 | s__Butyrivibrio_sp._AE2032 | X585 |
| s__Bacteroides_sp._CAG.189 | X286 | s__Pelosinus_fermentans | X586 |
| s__Clostridium_sp._CAG.253 | X287 | s__Anaerostipes_unclassified | X587 |
| s__Bacteroides_faecis_CAG.32 | X288 | s__Phascolarctobacterium_sp._CAG.266 | X588 |
| s__Ruminococcus_sp._CAG.17 | X289 | s__Lachnospiraceae_bacterium_oral_taxon_082 | X589 |
| s__Clostridium_sp._KNHs205 | X290 | s__Pseudobutyrivibrio_sp._MD2005 | X590 |
| s__Catonella_morbi | X291 | s__Eubacterium_sp._CAG.841 | X591 |
| s__Erysipelotrichaceae_bacterium_3_1_53 | X292 | s__Lachnospira_multipara | X592 |
| s__Lachnospiraceae_bacterium_5_1_63FAA | X293 | s__Pseudobutyrivibrio_sp._LB2011 | X593 |
| s__Coprobacillus_unclassified | X294 | s__Sutterella_parvirubra | X594 |
| s__Bacteroides_sp._CAG.598 | X295 | s__Butyrivibrio_sp._VCD2006 | X595 |
| s__Bacteroides_intestinalis_CAG.315 | X296 | s__Odoribacter_sp._CAG.788 | X596 |
| s__Clostridiaceae_bacterium_MS3 | X297 | s__Lachnospiraceae_bacterium_2_1_46FAA | X597 |
| s__Oscillibacter_ruminantium | X298 | s__Clostridium_perfringens | X598 |
| s__Clostridium_hathewayi_CAG.224 | X299 | s__Ruminococcus_sp._CAG.724 | X599 |
| s__Coprobacillus_sp._8_2_54BFAA | X300 | s__Ruminococcus_sp._CAG.353 | X600 |
