# Supplementary material for: Metagenomic study of the gut microbiota associated with cow milk consumption in Chinese peri-/postmenopausal women
Source: Front Microbiol. 2022 Aug 16;13:957885. doi: 10.3389/fmicb.2022.957885 (PMC9425034; doi:10.3389/fmicb.2022.957885)
Supplement: Supplementary file 6 [file Table_6.DOCX]

Supplementary Table 6 Characteristics of American cohort included in the study

| Variables | Control Group (n= 130) | CMC Group (n = 130) | *P* value |
| --- | --- | --- | --- |
| Female, n (%) * | 130 (100) | 130 (100) | 1.000 |
| Postmenopausal, n (%) * | 67 (51.5) | 69 (53.1) | 0.494 |
| Age (years; mean ± SD) # | 54.7 ± 13.8 | 55.3 ± 13.6 | 0.730 |
| Weight (KG; mean ± SD) # | 74.0 ± 18.0 | 72.3 ± 19.4 | 0.465 |
| BMI (mean ± SD) # | 27.7 ± 6.6 | 27.1 ±7.1 | 0.484 |
| Drinking, n (%) * | 48 (0.369) | 56 (43.1) | 0.374 |
| Exercise, n (%) * | 86 (66.1) | 95 (73.1) | 0.281 |

^*^Chi-squared test

^#^Two independent-sample *t* test
